# Kinetic Studies on the Interaction of HIV-1 Gag Protein with the HIV-1 RNA Packaging Signal

**DOI:** 10.3390/v16101517

**Published:** 2024-09-25

**Authors:** Constance Rink, Tomas Kroupa, Siddhartha A. K. Datta, Alan Rein

**Affiliations:** HIV Dynamics and Replication Program, National Cancer Institute, Frederick, MD 21702, USA; connie.rink@nih.gov (C.R.); tokroupa@gmail.com (T.K.); dattasi@mail.nih.gov (S.A.K.D.)

**Keywords:** HIV-1, packaging signal, Psi, protein–RNA interactions, binding kinetics

## Abstract

During HIV-1 virus assembly, the genomic RNA (vRNA) is selected for packaging by the viral protein Gag because it contains a specific packaging signal, Psi. While there have been numerous studies of Gag–Psi interactions, there is almost no information on the kinetic aspects of this interaction. We investigated the kinetics of Gag binding to different RNAs using switchSENSE DRX^2^ technology. We measured the association rate of Gag binding to monomeric Psi, to a “Multiple Binding Site Mutant” Psi that is inactive for genome packaging in vivo, and to a scrambled Psi. We discovered that Gag binds more rapidly to Psi RNA than to the mutant or scrambled RNAs. Furthermore, rapid Gag association kinetics are retained within sub-regions of Psi: Gag associates more rapidly with RNA containing only the 3′ two of the three Psi stem-loops than with monomeric RNA containing the 5′ two stem-loops or a scrambled RNA. No differences were detectable with individual Psi stem-loops. Interestingly, the rate of binding of Gag molecules to Psi increases with increasing Gag concentration, suggesting cooperativity in binding. The results are consistent with the hypothesis that selectivity in packaging derives from kinetic differences in initiation of particle assembly.

## 1. Introduction

Although much has been learned in recent years regarding the assembly of HIV-1 virus particles, the packaging of viral RNA is still only poorly understood. As the RNA is the material carrying the genetic information of the virus, it is essential that it is efficiently packaged into nascent virions during particle assembly. In fact, >90% of the particles in a typical virus preparation carry genomic RNA (vRNA) [1]. This selectivity of packaging depends upon the presence of the “packaging signal” (“Psi”), a highly structured region near the 5′ end of the vRNA [2,3,4]. The packaging signal is known to fold into three stem-loops known as SL1, SL2, and SL3 [5,6].

The HIV-1 virion is initially constructed from molecules of the Gag protein. Interestingly, when Gag is present in cells in which there is no RNA that contains Psi, it assembles into virus-like particles which contain cellular mRNA molecules in place of vRNA [7,8]. The individual mRNAs are packaged with almost no selectivity under these conditions [8]. These observations imply that vRNA is in competition with mRNAs for inclusion in the assembling virions, and that Psi provides the necessary advantage in this competition.

What is the nature of this advantage? One hypothesis is that Psi is a high-affinity binding site for Gag, but this hypothesis is not supported by experimental evidence, at least under physiological ionic conditions [9,10,11]. As an alternative explanation, we have proposed that Psi nucleates particle assembly more efficiently than RNAs lacking Psi [4,12,13].

In the present work, we have compared the rate at which recombinant Gag protein binds to Psi with its rate of binding to a control RNA. Using an experimental setup in which the two RNAs are presented simultaneously in a single flow cell, we find that it binds to Psi more rapidly than to the control. Intriguingly, the rate increases with increasing Gag concentration, implying that there is a cooperative element in the binding.

We also measured the kinetics of binding to individual stem-loops and pairs of Psi stem-loops. We observed that the Gag association kinetics are similar between the individual stem-loops of Psi, but differences in association kinetics appeared when Psi stem-loops were paired together. Our results imply that rapid Gag binding can be attributed to a specific subset of the stem-loop structures within Psi, but, apparently, not to any single stem-loop. Overall, our study implies that Gag binding kinetics could contribute to the selective packaging of the vRNA and provides additional knowledge on how the HIV-1 Gag interacts with nucleic acid.

## 2. Materials and Methods

### 2.1. Protein Expression and Purification

The expression and purification of the wild-type delta-p6 Gag was performed as previously described in [14]. 0.4 mM IPTG was used to overexpress delta-p6 Gag in *E. coli* BL21 (DE3) pLysS for 4 h at 37 °C. The bacterial lysate was treated with 0.1% (*w*/*v*) polyethylene imine to remove nucleic acids and then was subjected to 30% ammonium sulfate precipitation. The resulting pellet containing delta-p6 Gag was affinity-purified with phosphocellulose resin. To achieve a protein purity of >94%, size exclusion chromatography was subsequently performed using a Superose-12 HPLC column equilibrated with 20 mM HEPES (pH 7.4), 0.5 M NaCl, 2 mM DTT, and 10% *w*/*v* glycerol. The peak fractions of the delta-p6 Gag protein were pooled and concentrated to approximately 5 mg/mL. The purity of the protein was verified by SDS-PAGE and “Blue Silver” Coomassie staining before it was aliquoted into small volumes and stored at −80 °C. Before experiments, thawed aliquots were centrifuged at 14,000× *g* for 10 min at 4 °C to eliminate any generated aggregates.

### 2.2. Nucleic Acid Preparation 

The Psi sequences used in this study, i.e., monomeric Psi (nt 201-345); DIS sequence mutated to all C (DIS-6C); Multiple Binding Site mutant (DIS-6C with G224, G226, G240, G241, C243, G270, G272, G273, C274, G275, G289, G290, G292, G310, C312, G318, G320, G328, G239 mutated to A’s); and Scrambled Psi (nt 201-345), were all derived from the NL4-3 molecular clone. The Psi combination sequences were monomeric Psi 1-2 (DIS-6C) (nt 240-305), Psi 2-3 (nt 278-333), and Scrambled Psi 1-2 (scrambled nt 240-305). The individual stem-loops of Psi were SL1 (DIS-6C) (nt 243-277), SL2 (nt 278-305), SL3 (nt 304-333), and Scrambled SL3 (scrambled nt 301-335). All sequences mentioned above were cloned into the pUC19 backbone so that the resulting clones contained the T7 promoter, Psi sequence of interest, *Spe1* restriction site followed by 96 nucleotides complementary to A96 or B96 nanolever and *EcoR1* restriction site at the 3′ end of the transcripts. All plasmids were linearized with *EcoR1* and purified using the Nucleo spin kit (Macherey-Nagel, Duren, Germany). In vitro transcriptions were completed with the MEGAScript T7 kit (ThermoFisher Scientific, Waltham, MA, USA) following the vendor’s instructions. The RNA transcripts were purified using the Monarch RNA cleanup kit (New England BioLabs, Ipswich, MA, USA) and the RNAs eluted (30 μL) in nuclease-free water were stored at −80 °C. The yield of transcripts was determined by UV-Vis Nanodrop Lite (ThermoFisher Scientific). The integrity and ability of the RNAs to dimerize, and the lack of dimerization in Psi-DIS-6C RNA (Supplementary Figure S1), were confirmed by native gel electrophoresis (6% polyacrylamide/TBE). 

### 2.3. SwitchSENSE Kinetic Measurements

All kinetic measurements were performed on a DRX^2^ instrument (Dynamic Biosensors GmbH, Martinsried, Germany) using a two-colored MPC2-96-2-G1R1 biochip [15]. Before the kinetic measurements, the RNAs were pretreated at 95 °C for 3 min, fast-cooled on ice for 3 min, then diluted into minimal assay buffer (150 mM NaCl, 20 mM Tris (pH 7.5), 5 mM MgCl_2_), and incubated at 55 °C for 10 min. Prior to experiments, the biochips were treated with passivation solution (Dynamic Biosensors GmbH, Martinsried, Germany) containing a thiol-reactive compound to prevent non-specific binding of nucleic acid and protein on the chip surface. The RNA transcripts at 200 nM were then hybridized in the presence of PE40 buffer (10 mM NaPO_4_, 40 mM NaCl, 0.05% Tween 20, 50 μM EDTA, 50 μM EGTA, pH 7.4) at 37 °C to the ssDNA nanolevers (A96 and B96) on the surface and any excess RNA was washed away with degassed assay buffer (150 mM NaCl, 20 mM Tris (pH 7.5), 5 mM MgCl_2_, 1 mM TCEP, 0.03% Tween 20). For each association measurement, 400 μL of delta-p6 Gag (at concentrations of 31.2, 62.5, 125, or 250 nM) was injected at a flow rate of 200 μL/min at 25 °C. According to previous studies, the protein is almost entirely monomeric at these concentrations [16,17]. Dissociation of the protein was then measured with a flow rate of 200 μL/min of assay buffer. Following each Gag concentration tested, the bound RNAs were removed from the nanolevers with regeneration solution (pH 13) (Dynamic Biosensors GmbH, Martinsried, Germany).

### 2.4. Fitting of Kinetic Measurements

All DRX^2^ kinetic measurements were analyzed using GraphPad Prism (version 9.4.1) [18]. Kinetic data was fit to a least squares regression, One-phase association model equation as follows:

Y = Y0 + (Plateau − Y0)(1 − e^−Kx^)

where Y0 is the Y value when X (time) is zero. The plateau is the Y value at infinite times and K is the rate constant (k_obs_). This model was fitted individually to every Gag concentration and experimental replicate in this study (Supplementary Figures S2–S4).

## 3. Results

### 3.1. Psi RNAs Have Distinct Gag Association Kinetics Compared to Control RNA

**Figure 1:**
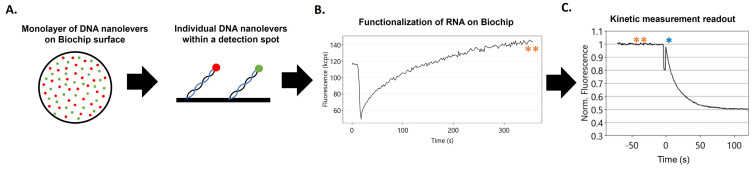
Workflow of a switchSENSE DRX^2^ experiment. The biochip surface has detection regions sparsely modified by DNA nanolevers. Each covalently tethered nanolever strand contains a fluorescent dye (red/B96 or green/A96) at the 3′ end and, before measurements, the complementary stabilizing DNA oligo sequence (cNL) shown in blue is removed (**A**). Functionalization/hybridization of the RNA transcripts on the biochip surface is achieved by hybridization to the single-stranded DNA nanolevers. The extent of hybridization of the RNA to the surface is monitored by the increase in nanolever fluorescence (orange Asterisk) (**B**). The kinetic measurement readout is a decrease in initial fluorescence (orange Asterisks) over time as the protein is flowed over the top (first addition of protein, blue Asterisks) (**C**). (An uncropped image of (**C**) is provided in the Supplementary Materials as Supplementary Figure S5).

We examined Gag–RNA binding kinetics by switchSENSE DRX^2^. This technology has been used to characterize a variety of protein–nucleic acid interactions [19,20,21]. The DRX^2^ instrument allowed for the simultaneous monitoring of protein association rates and dissociation rates from two different RNAs under identical conditions in a single flow cell. The measurements were performed in a microfluidic chamber within observation “spots” containing DNA nanolevers of two different sequences covalently attached to the chip surface [15]. In these experiments, RNAs containing, at their 3′ ends, a 96 nt sequence complementary to the DNA nanolever were hybridized to the monolayer of the DNA nanolever sequences on the DRX^2^ biochip surface (Figure 1A). The successful hybridization of RNAs with the DNA nanolevers on the biochip surface was followed by a change in fluorescence of the fluorophore at the 3′ end of each DNA nanolever (Figure 1B,C). The presence of different fluorophores (red or green) on the two “types” of nanolevers permitted the concurrent measurement of binding to different RNA molecules immobilized on each of these nanolevers. 

After the test RNAs were loaded onto the nanolevers, protein was flowed over the RNA-modified surface at a continuous rate, under controlled solvent conditions. The protein used in these experiments is recombinant HIV-1 Gag protein, differing from the authentic Gag protein in lacking a fatty-acid modification at its N-terminus and the p6 domain at its C-terminus [17,22]. Binding of Gag to an RNA on the chip surface alters the local environment of the fluorophore on the nanolever and quenches its fluorescence; this change in fluorescence is monitored over time after the addition of the Gag protein (Figure 1C). The release of Gag from the immobilized RNA is also measured during a period, in which the Gag solution is replaced with buffer alone in a “washout” step. After each run, the hybridized RNAs and any unbound proteins are stripped off, the RNA-decorated surface is regenerated, and a new binding reaction is performed.

**Figure 2:**
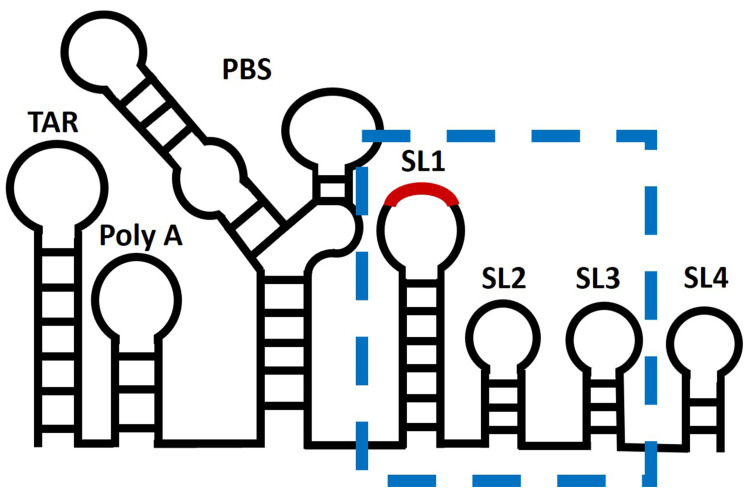
Model of the 5′ untranslated region of HIV-1 genome. The secondary structure model of the 5′UTR within the HIV-1 NL4.3 genome, nucleotides 1-356. Regions highlighted by a blue box are the part of the UTR used in this study, the packaging signal (Psi), which contains the stem-loops SL1, SL2, and SL3. The dimerization initiation site (DIS) within SL1 is represented in red.

Analysis of the resulting fluorescence traces yields parameters of association and dissociation. We compared Gag binding to three different RNA transcripts, i.e., monomeric Psi, Multiple Binding Site Mutant (MBSM) Psi, and scrambled Psi. The RNA transcripts are nucleotides 201-345 from the NL4-3 strain genome and contain elements of the packaging signal (SL1, SL2, SL3) (Figure 2). To simplify the analysis, we mutated the six-base palindromic sequence at the DIS of Psi to six C residues of DIS-6C to prevent dimerization of the RNA; this RNA is designated DIS-6C [23,24]. The MBSM (DIS-6C) RNA is a Psi mutant in which multiple G residues, important in Gag binding and in vRNA packaging in vivo [25,26,27], have been replaced with A residues. For a control RNA in our experiments, we chose to randomize the sequence of nucleotides 201-345 to ensure a similar nucleotide composition and length. 

In our experimental setup, we never observed dissociation of Gag from any of the RNAs at the washout step (Supplementary Figure S6). This is not due to loss of the fluorescent nanolevers (or the attached fluorophore) from the biochip surface, as fluorescence is recovered after stripping off any unbound protein and the RNAs during surface regeneration cycles. The fact that we could not detect dissociation of Gag during the washout step suggests that Gag binding to RNA at physiological salt is a stable interaction. This is consistent with the high affinity of Gag to all RNAs, as published previously [9,10] and confirmed in the present study using microscale thermophoresis (MST) (Supplementary Table S1). Based on these results, we chose to only discuss the data collected from the association phase of the kinetic measurements.

**Figure 3:**
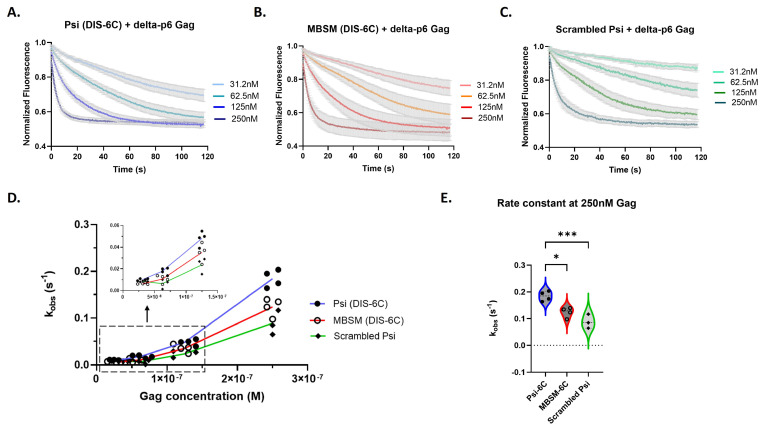
Psi RNAs have distinct Gag association kinetics compared to control RNA. Time-dependent response (normalized fluorescence) as a function of Gag concentration for monomeric Psi (**A**), Multiple Binding Site Mutant Psi (**B**), and scrambled control RNA (**C**). For each graph, the bold line(s) represents the mean response curve, and the light gray borders represent the SEM of three to four experiments. A line plot for each RNA showing the change in the observed association rate constant (k_obs_) with increasing Gag concentration (**D**); trends at the lowest Gag concentrations are highlighted within the dash lined box and inset graph. A violin plot comparing the association rates at 250 nM Gag (**E**), and analysis with one-way ANOVA (*** *p* < 0.001, * *p* < 0.05).

In our kinetic experiments, we observed a variation in association response curves, and we accurately show this range for all RNAs in Figure 3A–C. Due to the variation in the response curves, a global fitting method did not yield consistent rate constants. This led us to an analysis method of fitting each association response curve to a One-phase association model and deriving an observed association rate constant (k_obs_) for every protein concentration and experimental replicate (Supplementary Figure S2). To assess for trends, we plotted the data as k_obs_ versus Gag concentration. Overall, we found that Gag binds rapidly to all three RNAs (Figure 3A–C), with the fastest binding to monomeric Psi (DIS-6C) (Figure 3A). 

We also noted that the binding rate per Gag molecule increased with Gag concentrations, particularly above 125 nM, resulting in a “bend” in the graphed data (Figure 3D). The lack of linearity in these curves suggests that the binding of Gag to the RNAs is cooperative in the experimental setup at the concentration range tested (Figure 3D). In fact, only at 250 nM do we see Gag binding to monomeric Psi and MBSM (DIS-6C) RNA with a faster observed association rate constant than scrambled RNA (Figure 3D,E). At this concentration of Gag, binding to the MBSM (DIS-6C) RNA is slightly slower than monomeric Psi, suggesting that the specific unpaired G nucleotides mutated within MBSM (DIS-6C) contribute to rapid Gag binding kinetics (Figure 3E). 

### 3.2. The Individual Stem-Loops of Psi Have Similar Gag Association Kinetics

**Figure 4:**
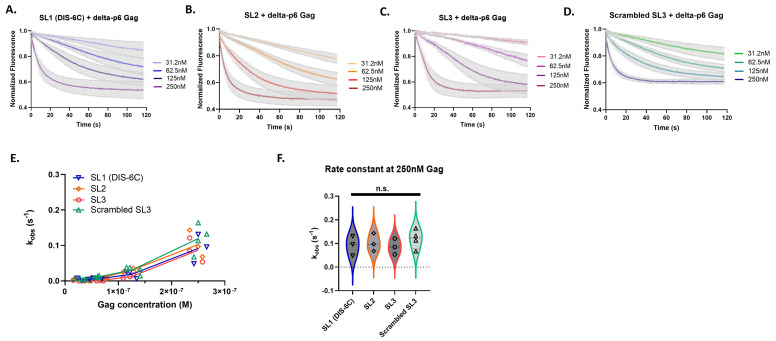
The individual stem-loops of Psi have similar Gag association kinetics. Time-dependent response (normalized fluorescence) as a function of Gag concentration for individual Psi stem-loops; monomeric SL1 (**A**), SL2 (**B**), SL3 (**C**), and scrambled control RNA (**D**). For each graph, the bold line(s) represents the mean response curve, and the light gray borders represent the SEM of three to four experiments. A line plot for each RNA showing the change in the observed association constant (k_obs_) with increasing Gag concentration (**E**). A violin plot comparing the association rates at 250 nM Gag (**F**), and analysis with one-way ANOVA (n.s., not significant).

We also investigated whether the Gag binding characteristics observed within the Psi sequence could be attributed to the individual stem-loops. We measured Gag binding to the individual stem-loops of Psi: monomeric SL1 (DIS-6C), SL2, SL3, and scrambled SL3. We followed the same analysis pipeline as described above for the longer RNA transcripts (Supplementary Figure S3). Remarkably the individual stem-loops of Psi, even though much smaller in length, retained similar characteristics to the long RNAs (145 nt), including the trend of non-linear kinetic rates (k_obs_) indicating cooperativity of binding (Figure 4). However, when we compared the association rates (k_obs_) amongst each of the individual stem-loops no significant differences were apparent, even at the highest Gag concentration (Figure 4F).

### 3.3. The Distinct Gag Association Kinetics Is Retained within Psi Stem-Loop Pairs

**Figure 5:**
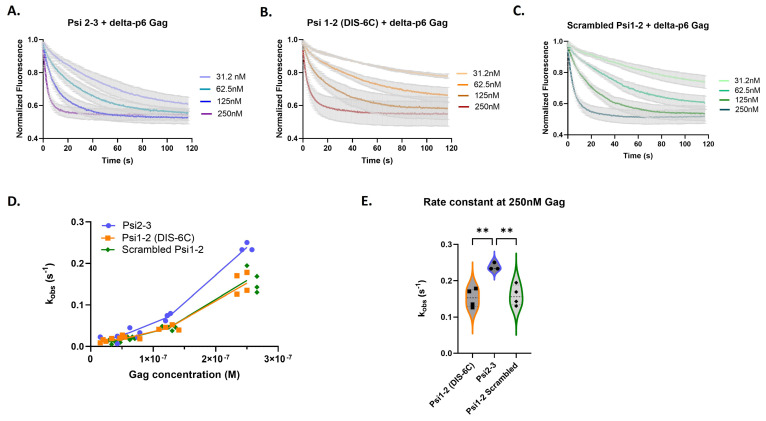
The distinct Gag association kinetics are retained within Psi stem-loop pairs. Time-dependent response (normalized fluorescence) as a function of Gag concentration for Psi stem-loops 2-3 (**A**), monomeric Psi stem-loops 1-2 (**B**), and scrambled control RNA (**C**). For each graph, the bold line(s) represent the mean response curve, and the light gray borders represent the SEM of three to four experiments. A line plot for each RNA showing the change in the observed association constant (k_obs_) with increasing Gag concentration (**D**). A violin plot comparing the association rates at 250 nM Gag (**E**), and analysis with one-way ANOVA (** *p* < 0.01).

We then investigated whether the rapid Gag binding to Psi could be localized to larger Psi fragments. We measured Gag association rates with RNAs containing two out of the three stem-loops of Psi: monomeric Psi 1-2 (DIS-6C), Psi 2-3, and scrambled Psi 1-2. We found (Figure 5) that Gag bound more rapidly to Psi 2-3 RNA than to the other RNAs (Figure 5D). Additionally, the rate constant for Psi 2-3 binding was higher at 250 nM Gag than at lower Gag concentrations (Figure 5D,E), just as was observed for the complete Psi RNA (Figure 3D). This result implies the existence of differential Gag binding kinetics within the packaging signal and reveals that quantifiable differences in Gag binding kinetics only arise when Psi stem-loops are paired together, suggesting that the structural organization of Psi might influence Gag binding kinetics. 

## 4. Discussion

How HIV-1 Gag protein selectively packages vRNA during virus particle assembly is not understood. The selection is, apparently, not due to a specific high binding affinity of the protein for the “packaging signal” (Psi) in the vRNA [9,10,11]. These studies showed that Gag binds with higher affinity to Psi than to control RNAs under conditions measuring specific binding, but that this special affinity is obscured by the high affinity of Gag for any RNA under physiological conditions. However, Gag binding kinetics have been suggested to play a role in the selection of the “packageable” conformation of the vRNA [28]. In the present work, we have measured the rate at which HIV-1 Gag protein binds to a stretch of the HIV-1 “packaging signal” located near the 5′ end of the genomic RNA. We found that Gag binds slightly faster to Psi than to a scrambled version of the same RNA. It was also striking to note that the rate of binding per Gag molecule is higher at a high Gag concentration than at a low Gag concentration, indicating a cooperative element in the interaction. A consistent trend we observed is that Gag binding at the lower concentrations, below 125 nM, fit well to a single-phase association model, but that the binding at an elevated concentration of 250 nM fit better to a two-phase association model (Supplementary Table S2). 

We also tested the possibility that the rapid binding could be attributed to a specific region within Psi. There are three stem-loops in Psi. We found no detectable differences in the binding to individual stem-loops (or to a scrambled RNA); thus, there is no indication of specificity with these small (~20–30-nucleotide) RNAs (Figure 4). However, binding to an RNA comprised of SL2 joined to SL3 was reproducibly faster than binding to an RNA consisting of SL1 and SL2, or to a scrambled RNA of similar length (Figure 5). It is interesting to note that the activity of Psi as packaging signal depends upon the relative positions of the stem-loops in the RNA [29]. Our results suggest that the three-dimensional organization of the stem-loops of Psi could contribute to the binding properties of Gag.

The kinetics of the interaction of Gag with RNAs are very likely an important factor in its ability to act as a nucleic acid chaperone [30,31,32] as, for example, in catalyzing the annealing of primer tRNA to genomic RNA during particle assembly [33,34,35,36]. In fact, Yang et al. reported that Gag binds more rapidly to a Psi RNA with a conformation promoting dimerization than to an alternative conformation [28]. To explain the selective packaging of Psi-containing RNA during virus assembly, we propose that Psi might represent a site at which assembly is initiated particularly efficiently [4,10,37]. It seems reasonable to imagine that the assembly of a virus particle occurs in two distinct phases, a slow, rate-limiting “initiation” or “nucleation” step, in which Gag molecules bind to naked or near-naked RNA, followed by a more rapid accretion of Gag molecules on the nascent lattice. This has been carefully documented in brome mosaic virus assembly [38], and the role of a packaging signal in nucleation has been considered in detail by Perlmutter and Hagan [39]. In this scenario, the identity of the encapsidated RNA is clearly determined at the initiation step. The switchSENSE experiments are an attempt to mimic this first step in assembly. The differences in rates we have observed are modest and might seem inadequate to explain selective packaging; however, we do not know how such differences might be compounded or enhanced during virus assembly. For example, it is conceivable that nucleation of particle formation depends upon the binding of multiple Gag molecules to a single RNA; in this hypothetical scenario, nucleation might proceed far more rapidly on Psi-containing RNA than on other RNAs.

It should be noted that in the switchSENSE technology, binding is detected by quenching of a fluorophore which is positioned at a specific site (the 3′ end of the DNA nanolever) that is adjacent to the 3′ end of the Psi segment being tested. Unfortunately, the contributions of Gag binding to distant regions of the RNA are not specifically resolved in the measured signal change. Further, we do not know how the binding of additional Gag molecules will affect the results. Because these uncertainties cannot be disentangled from each other, this is a major limitation of the information that we can obtain from these measurements. 

We have previously reported that Gag binds to RNAs in a cooperative manner [12]. In other words, Gag appears to have a higher affinity for a Gag–RNA complex than for naked RNA. This is consistent with the idea that binding to RNA is an essential first step in particle assembly, and that RNA can indeed provide a nucleation or initiation site for assembly. Our new data in conjunction with our previous study indicate that Gag binds not only with higher affinity, but also more rapidly to a Gag–RNA complex than to free RNA. 

The results show that Gag binds faster to Psi than to control RNAs, and appears to bind faster to a Gag–RNA complex than to free RNA. Enhanced binding to Gag–RNA complexes is a possible way of amplifying the rather modest differences in rates between binding to Psi and binding to other RNAs. Taken together, our results suggest that binding kinetics could contribute to the selective packaging of Psi-containing RNA. Furthermore, a recent paper [28] compared the rates at which Gag binds to two different RNAs, with conformations putatively like the alternative conformations of HIV-1 vRNA, using biolayer interferometry. These investigators found that Gag binds more rapidly to the “packageable” conformation than to the “translatable” conformation. These results also support the hypothesis that Gag binds uniquely and rapidly to packageable vRNA.

## Data Availability

The original contributions presented in this study are included in the article and Supplementary Materials; further inquiries can be directed to the corresponding author.

## Supplementary Materials

The following supporting information can be downloaded at https://www.mdpi.com/article/10.3390/v16101517/s1. Figures S1–S6; Tables S1 and S2; Supplementary Materials and Methods.
