# Supplementary material for: Kinetic Studies on the Interaction of HIV-1 Gag Protein with the HIV-1 RNA Packaging Signal
Source: Viruses. 2024 Sep 25;16(10):1517. doi: 10.3390/v16101517 (PMC11512386; doi:10.3390/v16101517)
Supplement: Supplementary file 1 [file viruses-16-01517-s001.zip › viruses-3170959-supplementary.pdf]

**Supplementary Materials**

*Supplemental Figures*

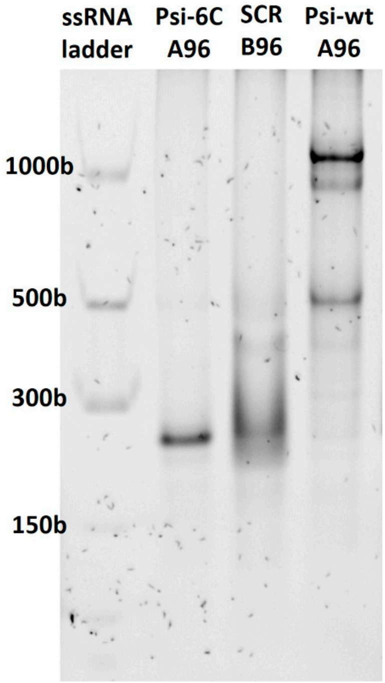


**Figure S1.** Assessment of conformation state of 145nt-length RNAs by native RNA gel electrophoresis. The Psi (DIS-6C) RNA containing the nanolever sequence remains monomeric after treatment with dimerization buffer and native gel electrophoresis (6% polyacrylamide/TBE) in the presence of 5 mM $MgCl_2$.

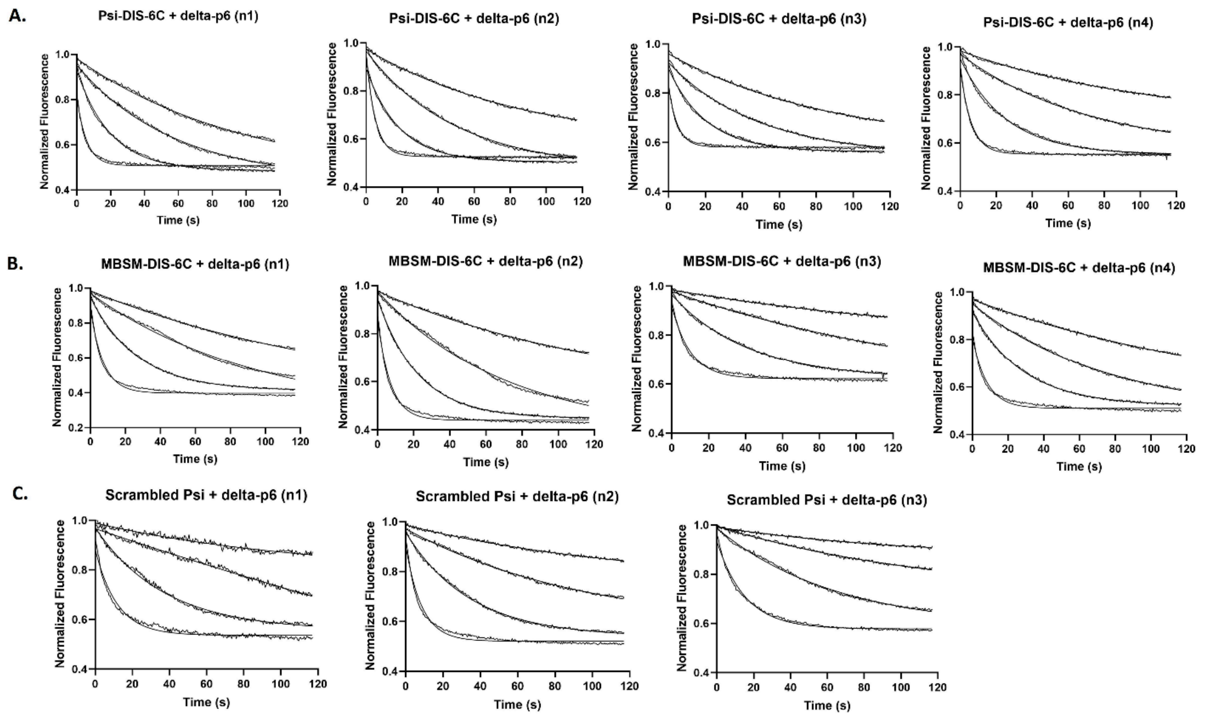


**Figure S2.** One-phase association model fit to each experimental replicate(s), 145-nt length RNAs: Psi (DIS-6C) (**A**), MBSM (DIS-6C) (**B**), Scrambled Psi (**C**). For every replicate (n) Gag concentration goes in 2-fold steps from 31.2 nM to 250 nM (top to bottom response curve).

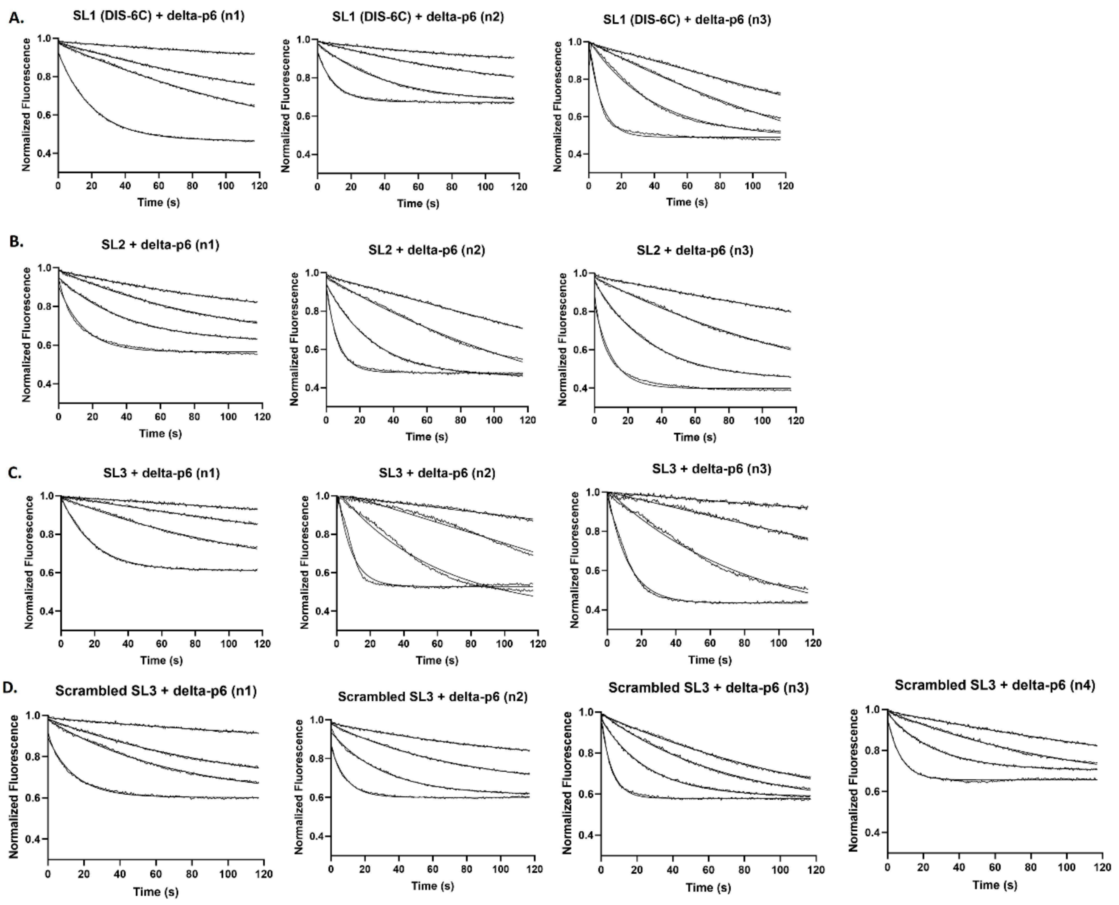


**Figure S3.** One-phase association model fit to each experimental replicate(s), stem-loop RNAs: SL1 (DIS-6C) (**A**), SL2 (**B**), SL3 (**C**), Scrambled SL3 (**D**). For every replicate (n) Gag concentration goes from 31.2 nM to 250 nM (top to bottom response curve).

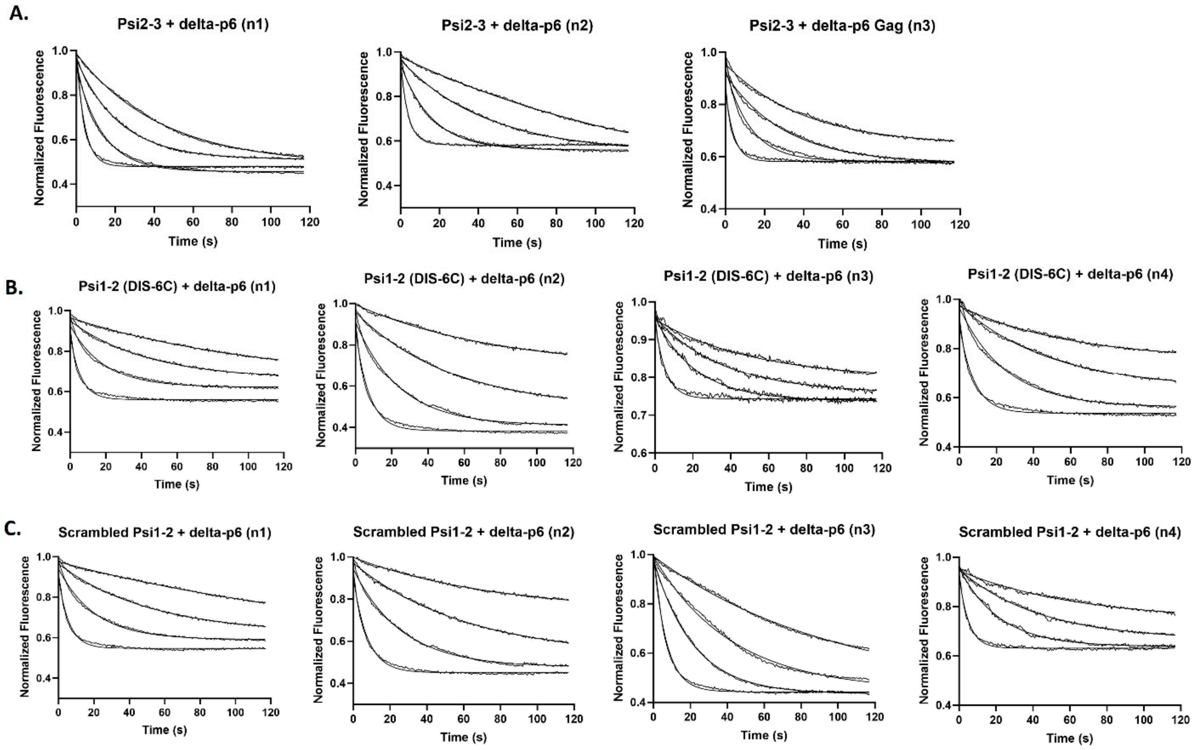


**Figure S4.** One-phase association model fit to each experimental replicate(s), stem-loop combination RNAs: Psi 2-3 (**A**), Psi 1-2 (DIS-6C) (**B**), Scrambled Psi (**C**). For every replicate (n) Gag concentration goes from 31.2 nM to 250 nM (top to bottom response curve).

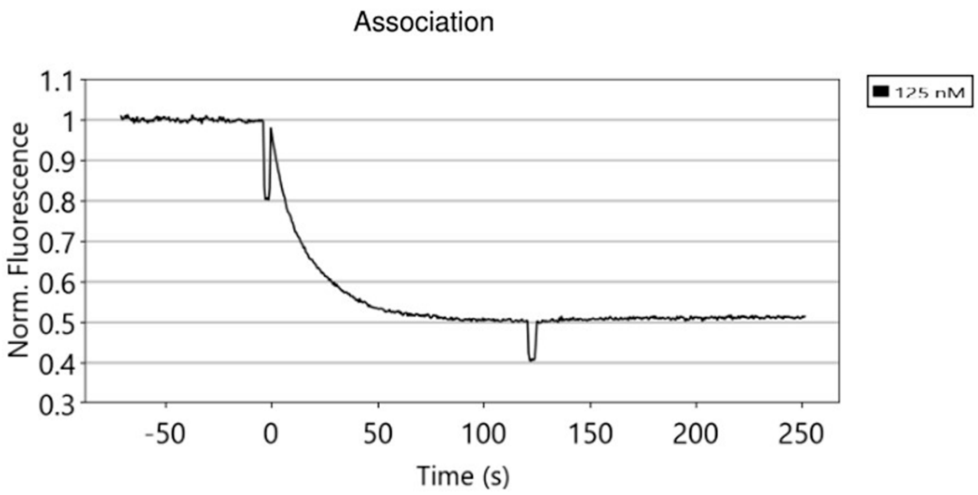


$k_{ON}$ = N/A

**Figure S5**. The raw association (~0-120 sec) and dissociation (~120-250 sec) curve for Psi (DIS-6C) at 125 nM of Gag.

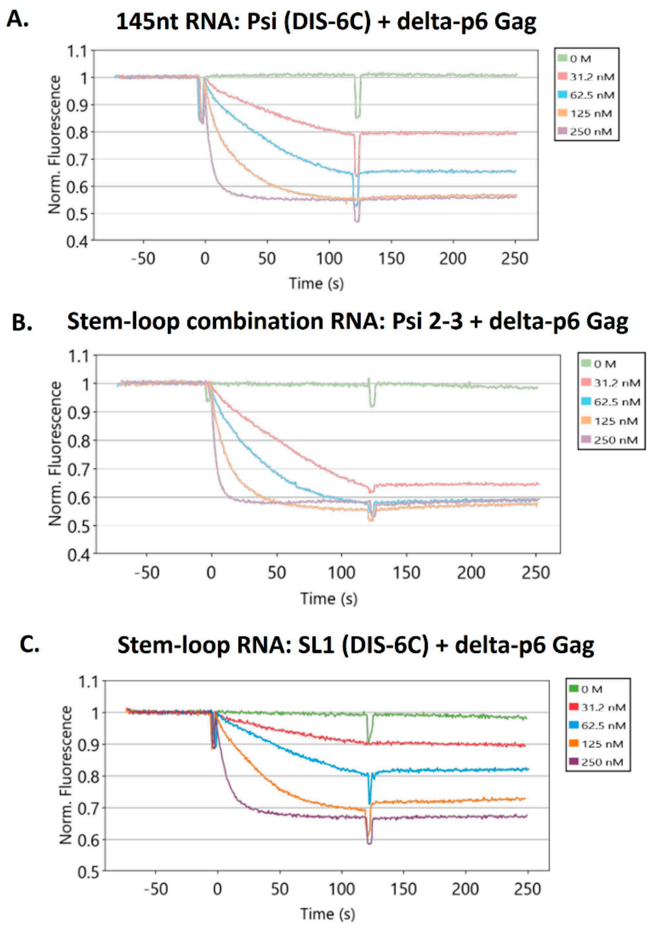


**Figure S6**. Single replicate examples of the raw association (~0-120 sec) and dissociation (~120-250 sec) curves for each of the RNA lengths (145nt (**A**), stem-loop combination (**B**), stem-loop (**C**)) tested in physiological salt buffer. No detectable dissociation of delta-p6 Gag is observed for any RNA length measured by switchSENSE.

**Table S1. Gag-RNA binding properties in 150mM salt buffer**

| RNA (145nt) | $K_D$ (nM) |
|---|---|
| Psi (DIS-6C) | 14.5 ± 3.74 |
| Scrambled Psi | 10.2 ± 2.09 |
| MBSM (DIS-6C) | 19.9 ± 3.85 |
| **RNA (stem-loop pairs)** | **$K_D$ (nM)** |
| Psi 1-2 (DIS-6C) | 14.3 ± 4.10 |
| Psi 2-3 | 12.3 ± 5.45 |
| Scrambled Psi (1-2) | 15.7 ± 1.82 |
| **RNA (stem-loop)** | **$K_D$ (nM)** |
| SL1 (DIS-6C) | 94.7 ± 22.2 |
| SL2 | 17.9 ± 6.84 |
| SL3 | 9.73 ± 2.43 |
| Scrambled SL3 | 101.4 ± 27.6 |

**Table S1.** Summary of Gag binding affinity measurements (MST) in physiological salt buffer for all RNA transcripts. Binding affinity values reported are representative of two to three experimental replicates.

## Table S2. Model fit comparison at 250 nM Gag (one-phase vs. two-phase association)

| RNA transcript | F test value | P-value |
|---|---|---|
| Psi (DIS-6C) | 422.9 | <0.0001 |
| Scrambled Psi | 552.0 | <0.0001 |
| MBSM (DIS-6C) | 1875 | <0.0001 |
| Psi 2-3 | 221.8 | <0.0001 |
| Psi 1-2 (DIS-6C) | 1984 | <0.0001 |
| Scrambled 1-2 | 589.7 | <0.0001 |
| SL1 (DIS-6C) | 165.8 | <0.0001 |
| SL2 | 908.0 | <0.0001 |
| Scrambled SL3 | 312.9 | <0.0001 |

**Table S2.** A statistical comparison of model fits to the highest Gag concentration for RNA transcripts. For each experimental replicate, the fit of one-phase association is compared with the fit of two-phase association model by extra sum-of-squares F test. The extra sum-of-squares F test for one of the experimental replicates is reported, representing the overall trend of data. The large F test value(s) and p-value(s) indicate that a two-phase association model fits the data significantly better than a one-phase association model.

**Supplemental Material and Methods**

*Dimerization treatment of RNA and native gel electrophoresis*

The RNA transcripts (1.0 ug) were heated to 95°C for 3 minutes, fast-cooled on ice for 3 minutes, followed by addition of dimerization buffer (300 mM KCl, 50 mM Tris (pH 7.5), 5 mM $MgCl_2$) and incubated at 55°C for 30 minutes (protocol modified from [10]). The extent of dimerization was verified by native gel electrophorsis (6% polyacrylamide/TBE). The native gel electrophorsis was carried out at room temperature at 110V with 1x-TBE running buffer supplemented with 5 mM $MgCl_2$ and followed by staining with SYBR safe DNA gel stain (ThermoFisher Scientific, Waltham, MA, USA).

*RNA preparation and RNA labeling for thermophoresis*

RNA transcripts were prepared as described in the main section of the publication with the following modification. The plasmids were digested with *Spe1* to ensure exclusion of the 96-nts nanolever sequence from the transcripts. Following in vitro transcription and purification, the RNAs were 3'-end Cy5 labeled with pCp-Cy5 (Jena Bioscience GmbH, Jena, Germany) as previously described [10].

*Microscale thermophoresis (MST) measurements*

MST measurements were performed as previously described [11] in premium coated capillaries on a Monolith NT. 115 instrument (Nanotemper Technologies GmbH, Munich, Germany). All measurements were completed in the buffer containing 150 mM NaCl, 20 mM Tris (pH 7.5), 1 µM $ZnCl_2$, 5 mM $MgCl_2$, 0.05% Tween20, 0.1 mM phenylmethylsulfonylfluoride (PMSF), and 1 mM β-mercaptoethanol (βME). The calculation of Kd values for all measurements were performed using the MO. Affinity Analysis software provided by the manufacturer (v2.1.3).

*Model fit comparison*

The statistical comparison of model fits to the highest Gag concentration was performed using the program GraphPad Prism (version 9.4.1). The association measurements at 250 nM of Gag were fit to both One-phase association model equation (1) and Two-phase association model (2):

(1). $Y=Y0 + (Plateau-Y0)(1-e^{-Kx})$

(2). $Y=Y0 + (SpanFast)(1-e^{-KFast(x)}) + (SpanSlow)(1-e^{-Kslow(x)})$

$SpanFast=(Plateau-Y0)(\%Fast)(0.1)$

$SpanSlow=(Plateau-Y0)(100-\%Fast)(0.1)$

The extra sum-of-squares F test equation was used to compare the fits of the two models:

$$F=\frac{(SS1-SS2)/(DF1-DF2)}{SS2/DF2}$$

Where SS1 is the sum-of-squares for model (1) and SS2 is the sum-of-squares for model (2). The DF1 is the degrees of freedom for model (1) and DF2 is the degrees of freedom for model (2).
